# Supplementary material for: Genomic insights into antibiotic resistance and mobilome of lactic acid bacteria and bifidobacteria
Source: Life Sci Alliance. 2023 Feb 13;6(4):e202201637. doi: 10.26508/lsa.202201637 (PMC9930590; doi:10.26508/lsa.202201637)
Supplement: Supplementary file 8 [file LSA-2022-01637_TableS8.docx]

**Supplementary Table S8 - The BLAST alignment of the resistance genes database against a test dataset SwissProt (EMBL-EBI, United Kingdom) at different similarity cut-offs.** **(A)** Positive predictive value (PPV), negative predictive value, sensitivity, and specificity were calculated. **(B)** The ROC curve shows a plot of the true positive rate (sensitivity) in function of the false positive rate (1-specificity). **(C)** The PR curve shows a plot of the PPV in function of true positive rate (sensitivity).

A.

| **Similarity cut-off** | **Positive** | **True positive** | **False positive** | **Negative** | **True Negative** | **False negative** | **Positive predictive value** | **Sensitivity** | **1-specificity** | **Specificity** | **Negative predictive value** |
| --- | --- | --- | --- | --- | --- | --- | --- | --- | --- | --- | --- |
| 90 % | 944 | 936 | 8 | 563694 | 561715 | 1979 | 99.15 % | 32.11 % | 0.00 % | 100.00 % | 99.65 % |
| 80 % | 1146 | 1134 | 12 | 563492 | 561711 | 1781 | 98.95 % | 38.90 % | 0.00 % | 100.00 % | 99.68 % |
| 70 % | 1309 | 1283 | 26 | 563329 | 561697 | 1632 | 98.01 % | 44.01 % | 0.00 % | 100.00 % | 99.71 % |
| 60 % | 1747 | 1671 | 76 | 562891 | 561647 | 1244 | 95.65 % | 57.32 % | 0.01 % | 99.99 % | 99.78 % |
| 50 % | 2517 | 2374 | 143 | 562121 | 561580 | 541 | 94.32 % | 81.44 % | 0.03 % | 99.97 % | 99.90 % |

B.

C.
